# Influence of obesity on serum levels of SARS-CoV-2-specific antibodies in COVID-19 patients

**DOI:** 10.1371/journal.pone.0245424

**Published:** 2021-03-24

**Authors:** Daniela Frasca, Lisa Reidy, Carolyn Cray, Alain Diaz, Maria Romero, Kristin Kahl, Bonnie B. Blomberg

**Affiliations:** 1 Department of Microbiology and Immunology, University of Miami Miller School of Medicine, Miami, FL, United States of America; 2 Sylvester Comprehensive Cancer Center, University of Miami Miller School of Medicine, Miami, FL, United States of America; 3 Department of Pathology & Laboratory Medicine, University of Miami Miller School of Medicine, Miami, FL, United States of America; Emory University School of Medicine, UNITED STATES

## Abstract

SARS-CoV-2 (Severe Acute Respiratory Syndrome Corona Virus-2), cause of COVID-19 (Coronavirus Disease of 2019), represents a significant risk to people living with pre-existing conditions associated with exacerbated inflammatory responses and consequent dysfunctional immunity. In this paper, we have evaluated the influence of obesity, a condition associated with chronic systemic inflammation, on the secretion of SARS-CoV-2-specific IgG antibodies in the blood of COVID-19 patients. Our hypothesis is that obesity is associated with reduced amounts of specific IgG antibodies. Results have confirmed our hypothesis and have shown that SARS-CoV-2 IgG antibodies are negatively associated with Body Mass Index (BMI) in COVID-19 obese patients, as expected based on the known influence of obesity on humoral immunity. Antibodies in COVID-19 obese patients are also negatively associated with serum levels of pro-inflammatory and metabolic markers of inflammaging and pulmonary inflammation, such as SAA (serum amyloid A protein), CRP (C-reactive protein), and ferritin, but positively associated with NEFA (nonesterified fatty acids). These results altogether could help to identify an inflammatory signature with strong predictive value for immune dysfunction. Inflammatory markers identified may subsequently be targeted to improve humoral immunity in individuals with obesity and in individuals with other chronic inflammatory conditions.

## Introduction

SARS-CoV-2 (Severe Acute Respiratory Syndrome Corona Virus-2), the cause of COVID-19 (Coronavirus Disease of 2019), has been efficiently spreading from human-to-human since the last months of 2019 and has been responsible for mild-to-severe respiratory tract infections. Our knowledge of human immune responses to SARS-CoV-2 infection is limited, and the host factors responsible for disease progression and symptom severity are largely unknown. Recently published data have indicated that chronic low-grade systemic inflammation, inflammaging [1], is the major cause of the cellular and molecular changes induced by SARS-CoV-2 and is responsible for the highest mortality rates [2]. Inflammaging has been shown to induce chronic immune activation (IA) associated with impairment of immune cell function, as reviewed in [3].

Viral clearance and resolution of SARS-CoV-2 infection requires a complex immune response initiated by resident epithelial cells and innate immune cells, followed by adaptive immune cells that effectively cooperate to eliminate the virus. B cells contribute to viral clearance by producing virus-specific antibodies that can neutralize the virus, thus preventing the spread of infectious virions, controlling virus dissemination, and reducing tissue damage. Previously published data have demonstrated strong neutralizing antibody responses generated against the Spike glycoprotein of the SARS-CoV of the 2002–2003 pandemic protected infected hosts from severe disease [4]. Moreover, it has been postulated that during the current pandemic, the production of antibodies to SARS-CoV-2 is critical to limit disease progression, and neutralizing antibodies present in plasma from convalescent COVID-19 patients have been shown to induce fast recovery of critically ill patients [5–7].

Obesity, like viral infections, induces persistent local and systemic inflammation and chronic IA, contributing to functional impairment of immune cells, and decreased immunity. Obesity and associated inflammation lead to several debilitating chronic diseases such as type-2 diabetes, cancer, atherosclerosis, and inflammatory bowel disease [8–15]. Therefore, obesity has been identified as an additional risk factor for COVID-19 patients [16–19]. Also, the prevalence of the disease, and the occurrence of complications in obese individuals are increased compared to lean controls. Indeed, a strong association has been shown between obesity, obesity-associated comorbidities and severe outcomes of COVID-19 [20]. Retrospective analyses of adult COVID-19 symptomatic patients have demonstrated that individuals with Body Mass Index (BMI) >30 were more likely to be admitted to acute and critical care compared to individuals with a BMI <30 [21]. Previously, it has been demonstrated that obese patients respond poorly to infections [22–24], vaccination [25–27], and therapies [28]. The obesity-associated dysregulation of the immune system may also extend the duration and heighten the magnitude of the metabolic stress. It is well known that the obese adipose tissue (AT) is heavily infiltrated with immune cells [29, 30], which fuel local inflammation and exacerbate inflammaging. AT in the thorax and abdominal areas induce secretion of additional pro-inflammatory mediators that can further compromise lung function [31, 32]. The infiltrating immune cells once activated following SARS-CoV-2 infection, contribute to the release of inflammatory mediators. Another significant health problem is that the AT may be a viral reservoir, playing a crucial role in maintaining local and systemic inflammation, persistent IA, and immune dysfunction [33].

In this study, we have measured serum levels of SARS-CoV-2 Spike-specific IgG antibodies in lean and obese COVID-19 patients as well as in uninfected controls, using an ELISA test developed and standardized in our laboratory. Results obtained have confirmed our hypothesis that obesity and serum levels of SARS-CoV-2 Spike-specific IgG antibodies are negatively associated. Briefly, we show that higher BMI is associated with a higher infection rate with SARS-CoV-2, measured by serum detection of viral RNA and antibodies. Spike-specific IgG antibodies in obese individuals are negatively associated with BMI and serum levels of pro-inflammatory and metabolic markers of inflammaging and pulmonary inflammation. Results obtained have allowed the identification of inflammatory markers of disease with a strong predictive value for immune dysfunction that can be targeted to improve humoral immunity in COVID-19 patients with obesity.

## Materials and methods

### Enrolled participants

Experiments were performed using serum samples isolated from individuals tested negative or positive for SARS-CoV-2 RNA by reverse transcriptase-polymerase chain reaction (RT-PCR) of nasopharyngeal swab samples and by LFIA using the Healgen LFD with specificity for the SARS-CoV-2 Spike antigen (#GCOV-402a, CEHealgen LLC, Texas, USA). In total, 52 negative and 72 positive serum samples were collected from both inpatient and outpatient settings. All samples were from patients that presented to the University of Miami Health facilities (Miami, FL) for clinical care. There was no recruitment of patients for this study. These were discarded clinical samples, and there were no exclusion criteria other than that the patient had to be >18 years of age. Inclusion criteria for a positive sample was a clinical discarded serum sample with an RT-PCR performed within 24 hrs of sample collection. Samples were collected between 03/2020 and 08/2020, stored refrigerated (2-8°C) for up to 5 days and then maintained at -80°C. Samples were de-identified before use in this study.

The demographics, BMI, clinical test results, and clinical characteristics of the participants, and their SARS-CoV-2-specific IgG ELISA results, are shown in Table 1. Briefly, patients were older than uninfected controls and were 40/72 males and 32/72 females. Patients also had higher BMI as compared to uninfected controls, suggesting that the recruited participants in our study represent a larger population of individuals with obesity. No different counts of total WBC, neutrophils or lymphocytes were observed between patients and uninfected controls. Patients had comorbidities such as lung disease (14/72), type-2 diabetes mellitus (23/72), coronary heart disease (12/72), autoimmune disease (7/72), cancer (9/72), infectious disease (1/72). This research was approved and reviewed by the Institutional Review Board (IRB, protocol #20200504) at the University of Miami, which reviews all human research conducted at the University of Miami auspices.

**Table 1:** Demographics, laboratory and clinical characteristics of enrolled participants.

	Negatives (n = 52)	Positives (n = 72)
Age, mean±SE	33±3	65±2, p<0.0001
BMI, mean±SE (range)	23.5±1.4 (17.4–33.4)	27.7±2.0 (19.4–45.6), p = 0.0002
Males	15	40
Females	37	32
Spike-specific IgG (OD)	0.64±0.07	1.43±0.28, p<0.0001
Total WBC (x10^3^/μl)^	7.22±0.76	8.56±0.54
Neutrophils (x10^3^/μl)^^	4.85±0.99	6.16±0.52
Lymphocytes (x10^3^/μl)^^^	1.49±0.24	1.36±0.09
Chronic conditions/diseases		
Lung disease	0	14^a^
Type-2 Diabetes Mellitus	1	23
Coronary Heart disease	0	12
Autoimmune disease	0	7^b^
Cancer	2^c^	9^d^
Infectious disease	0	1^e^

^Normal values: 3.8–10.8 x10^3^/μl

^^Normal values: 2.5-8x10^3^/μl

^^^Normal values: 1–4.8 x10^3^/μl

^a^Lung disease patients: COPD (9), Hypersensitivity Lung disease (5)

^b^Autoimmune disease patients: Type-1 Diabetes (2), Parkinson’s (1), Alzheimer’s/dementia (3), Addison (1)

^c^Cancer patients: leukemia (2)

^d^Cancer patients: breast cancer (2), lung cancer (4), sarcoma (1), vulvar cancer (2)

^e^Infectious disease patients: hepatitis (1)

Both SARS-CoV-2 RT-PCR and serology tests were performed at Department of Pathology & Laboratory Medicine clinical laboratories. SARS-CoV-2 RT-PCR was performed at the University of Miami Hospital using either the Diasorin or the BDMax assay and reagents. Depending on submission type (symptomatic or asymptomatic) the sample was assigned and tested per manufacturer guidelines.

The Healgen LFD serology test was performed according to the manufacturer guidelines. To each cassette, 5 μL of serum was added, and two drops of the manufacturer-provided buffer solution were applied to the cassette immediately after the sample was loaded. Results were read 10 min after. The Healgen LFD were single-channel flow devices, with IgG, IgM, and C (control line) annotated on the cassettes. A pink line in either/or both IgG and IgM was recorded. Because the LFD results are not quantitative, only showing the presence or absence of specific IgM/IgG antibodies, we developed a Spike-specific ELISA (see below) to compare anti-Spike values with the other measures.

### ELISA to measure Spike-specific IgG antibodies

Serum IgG antibodies against SARS-CoV-2 Spike protein were measured by an ELISA developed and standardized in our laboratory. Briefly, 96-well microplates (Immulon 4HBX, Thermo Scientific) were coated with recombinant NCP-CoV (2019-nCoV) Spike protein (S1+S2 ECD) (Sino Biological #40589-V08B1) at 2 μg/mL for 1 hr at room temperature. Plates were then washed with Tween-20 0.05% in PBS (PBST) and blocked with assay buffer (1% BSA in PBS) for 1 hr at 37°C. After blocking, all subsequent steps were performed by a DYNEX DS2® Automated ELISA system (DYNEX Technologies, Chantilly, VA, USA). Serum samples diluted 1:50,000 in assay buffer were added in duplicate, and plates were incubated for 2 hrs. Plates were washed with PBST and 100 μL per well of a peroxidase-conjugated goat anti-human IgG (Jackson ImmunoResearch #109-036-098), diluted 1:10,000 in assay buffer, were added. After 1 hr incubation, plates were washed, and a stabilized 3,3′,5,5′-Tetramethylbenzidine (TMB) substrate (Sigma) was added to the wells. The enzymatic reaction was stopped after 20 min with a Stop solution (1 M sulfuric acid), and absorbance at 450 nm was read by the DYNEX DS2 instrument.

### ELISA to measure serum pro-inflammatory and metabolic markers

Serum levels of SAA, CRP, ferritin, and NEFA were measured using the following commercially available kits. SAA: Life Diagnostic #SAA-20. CRP: R&D # DCRP00. Ferritin: Thermo Scientific #EHFTL. NEFA: Abcam #ab65341.

### Statistical analyses

To examine differences between groups, unpaired Student’s t-tests (two-tailed) were used. To examine relationships between variables, bivariate Pearson’s correlation analyses were performed, using GraphPad Prism version 8 software, which was used to construct all graphs.

## Results

### Evaluation of Spike-specific IgG antibodies in the sera of study participants using an ELISA standardized in our laboratory

The test group consisted of 52 negative individuals, and 72 individuals tested positive for SARS-CoV-2 RNA detection by RT-PCR of nasopharyngeal swab samples and serum antibody-positive by lateral flow immunoassay (LFIA) using the lateral flow device (LFD). Age, gender, BMI, routine clinical laboratory measures, and the recruited participants’ chronic conditions and diseases are shown in Table 1. Despite several initial reports indicating that COVID-19 patients were characterized by significant lymphocytopenia [34], this cohort had normal numbers of total WBC, neutrophils, and lymphocytes, as also recently shown by other groups [35–37]. In addition to previously measured RT-PCR and LFD results, we also measured IgG antibodies specific for the SARS-CoV-2 (2019-nCoV) S1+S2 (Spike) recombinant protein, using an ELISA developed and standardized in our laboratory. This ELISA confirmed the results previously obtained with RT-PCR and LFD as all the individuals tested negative or positive by RT-PCR and LFD were also negative or positive in our Spike-specific ELISA, respectively. Results show significantly higher levels of Spike-specific IgG antibodies in positive *versus* negative individuals in our assay. Because there is a significant difference between the age of negative and positive individuals, we compared IgG levels in <65 year old (n = 29) and >65 year old (n = 43) positive individuals to rule out possible effects of aging. We found no significant differences between the two groups (1.3±0.1 and 1.5±0.2, respectively). Also, due to the higher frequency of male *versus* female positive individuals in this cohort (56% *versus* 44%), we compared IgG levels in the two groups, but no significant differences were found (1.5±0.2 *versus* 1.3±0.1, respectively, p = 0.32).

### BMI is higher in SARS-CoV-2 positive *versus* negative individuals and is associated with severe respiratory symptoms

**Fig 1:**
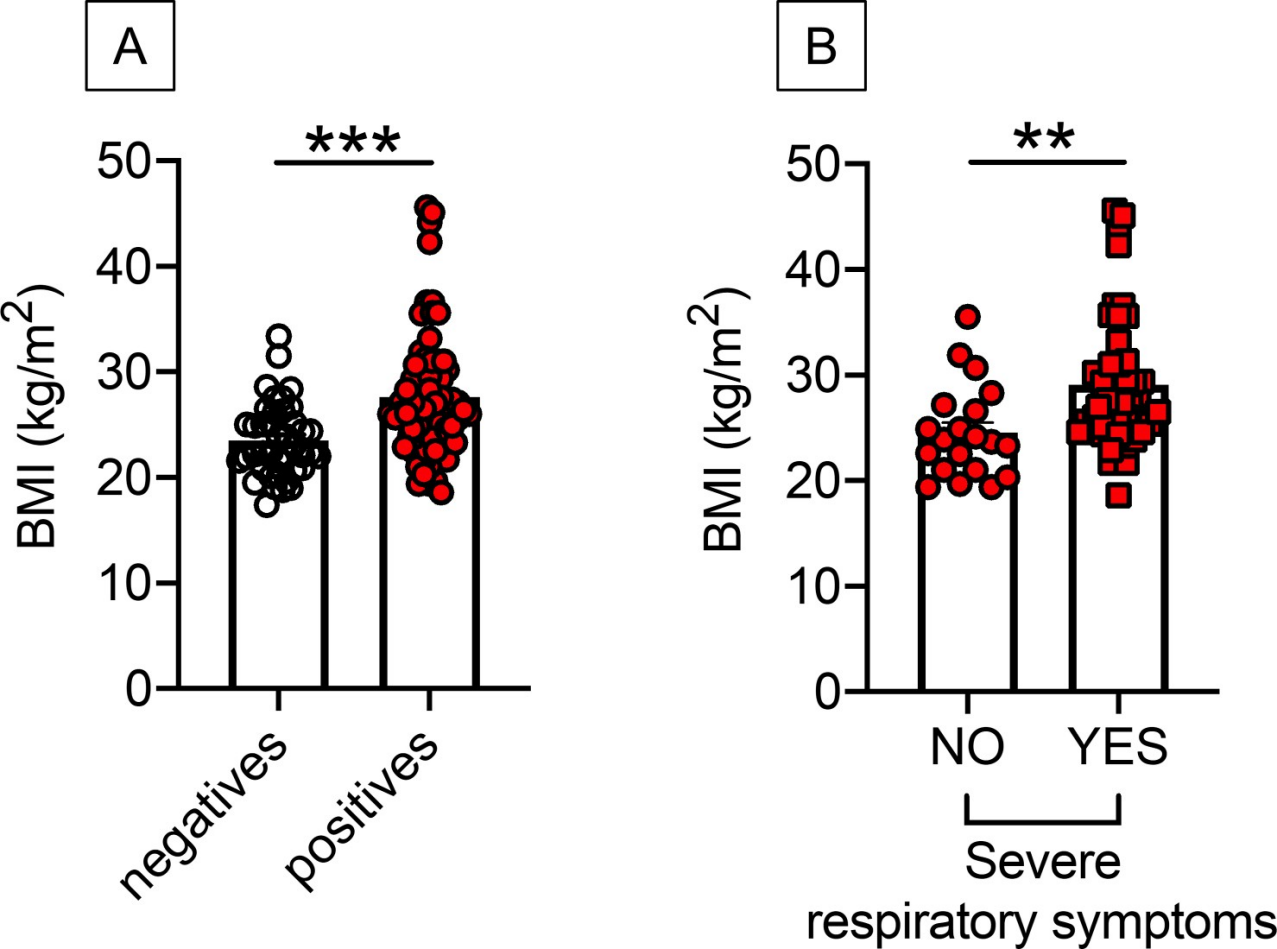
BMI is higher in positive *versus* negative individuals and is associated with severe respiratory symptoms. **A.** BMI in participants tested negative and positive by Spike-specific ELISA. **B.** BMI in COVID-19 positive patients with (n = 46) or without (n = 20) severe respiratory symptoms. Severe respiratory symptoms included high fever, cough, shortness of breath, hypoxia, as determined at the time of hospital admission. **p<0.01, ***p<0.001.

Next, we examined the BMI of positive and negative individuals. Results in Fig 1A show that BMI was higher in positive *versus* negative individuals (27.7 *versus* 23.5, respectively), suggesting a higher frequency of infected patients in obese *versus* lean individuals. In the group of positives, higher BMI was also associated with severe respiratory symptoms (29.1 *versus* 24.6, respectively), as evaluated at the time of hospital admission (Fig 1B). Severe respiratory symptoms were determined by physician evaluation at hospital admission and included high fever, cough, shortness of breath, and hypoxia. As expected, in positive individuals, leptin, the pro-inflammatory adipokine secreted primarily by the adipocytes and marker of obesity [38, 39], was found higher in obese *versus* lean individuals (3536±636 pg/ml *versus* 7852±634 pg/ml, p<0.0001).

### SAA, CRP and ferritin, markers of COVID-19, are higher in positive *versus* negative individuals and are associated with severe respiratory symptoms

**Fig 2:**
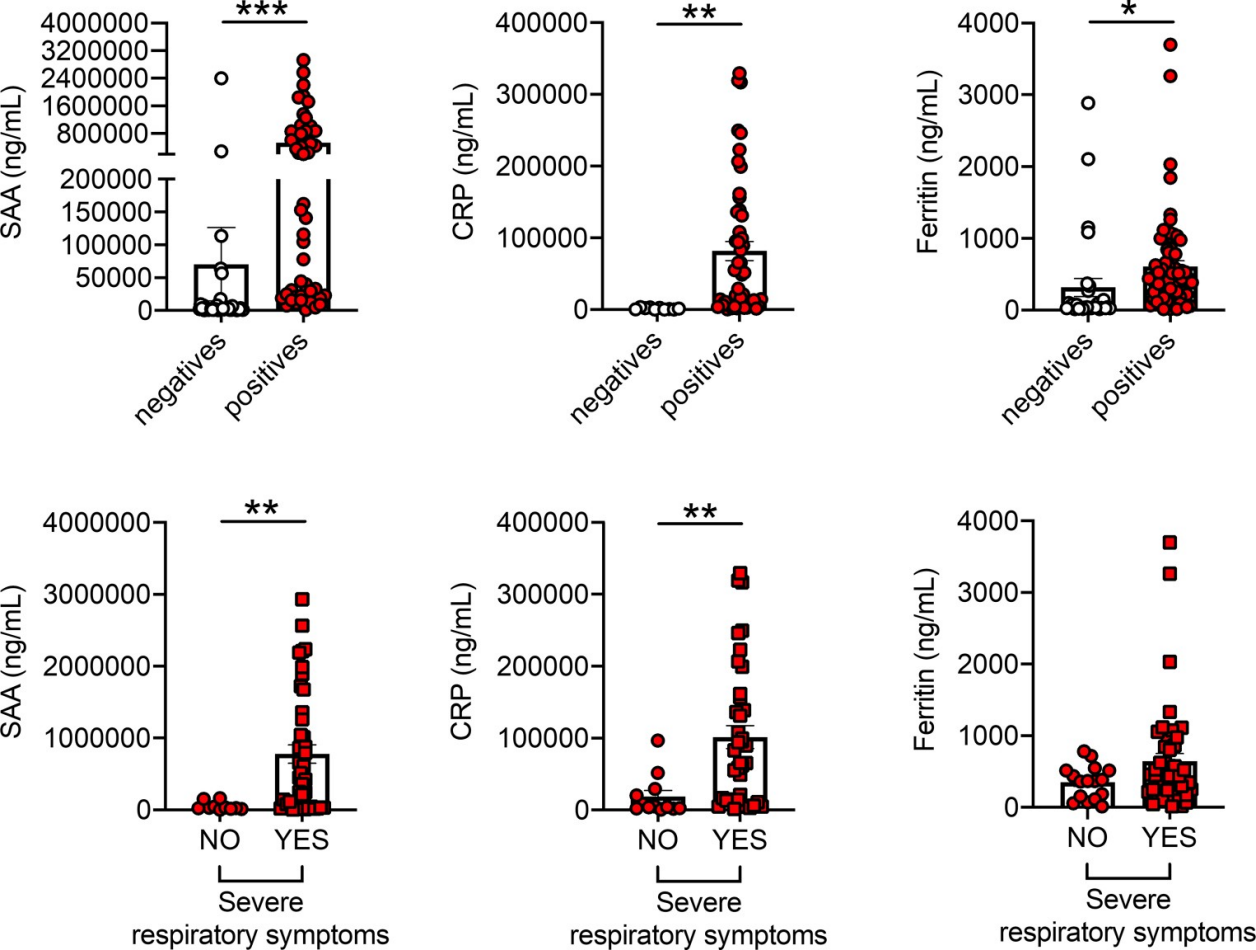
SAA, CRP and ferritin, markers of COVID-19, are higher in positive *versus* negative individuals and are associated with severe respiratory symptoms. **Top.** SAA, CRP and ferritin were detected in serum samples of participants who tested negative and positive. **Bottom.** SAA, CRP and ferritin in serum samples of COVID-19 positive patients with [n = 42 (SAA), n = 38 (CRP), n = 48 (ferritin)] or without [n = 10 (SAA), n = 10 (CRP), n = 16 (ferritin)] severe respiratory symptoms (as defined in Fig 1). *p<0.05, **p<0.01, ***p<0.001.

We measured pro-inflammatory and metabolic markers associated with inflammation in serum samples from positive and negative individuals. We measured SAA (serum amyloid A protein) [40, 41], CRP (C-reactive protein) [42–45] and ferritin [44, 46, 47]. These proteins are markers of pathogen-driven pulmonary inflammation, embolism, and disseminated intravascular coagulation, all characteristics of COVID-19, and therefore predictors of adverse health outcomes. Results in Fig 2, top show significantly higher levels of these markers in the serum of positive *versus* negative individuals. In the group of positives, SAA and CRP values were also higher in patients with severe respiratory symptoms than those without symptoms, whereas ferritin showed a trend borderline of significance (p = 0.06) (Fig 2, bottom). Levels of SAA, CRP and ferritin were found comparable in <65 year old and >65 year old positive individuals with severe respiratory symptoms (865,815±164,620 and 745,497±150,653, p = 0.56 for SAA; 113,087±118,121 and 88,221±18,422, p = 0.52 for CRP; 883±174 and 493±73, p = 0.08 for Ferritin).

**Fig 3:**
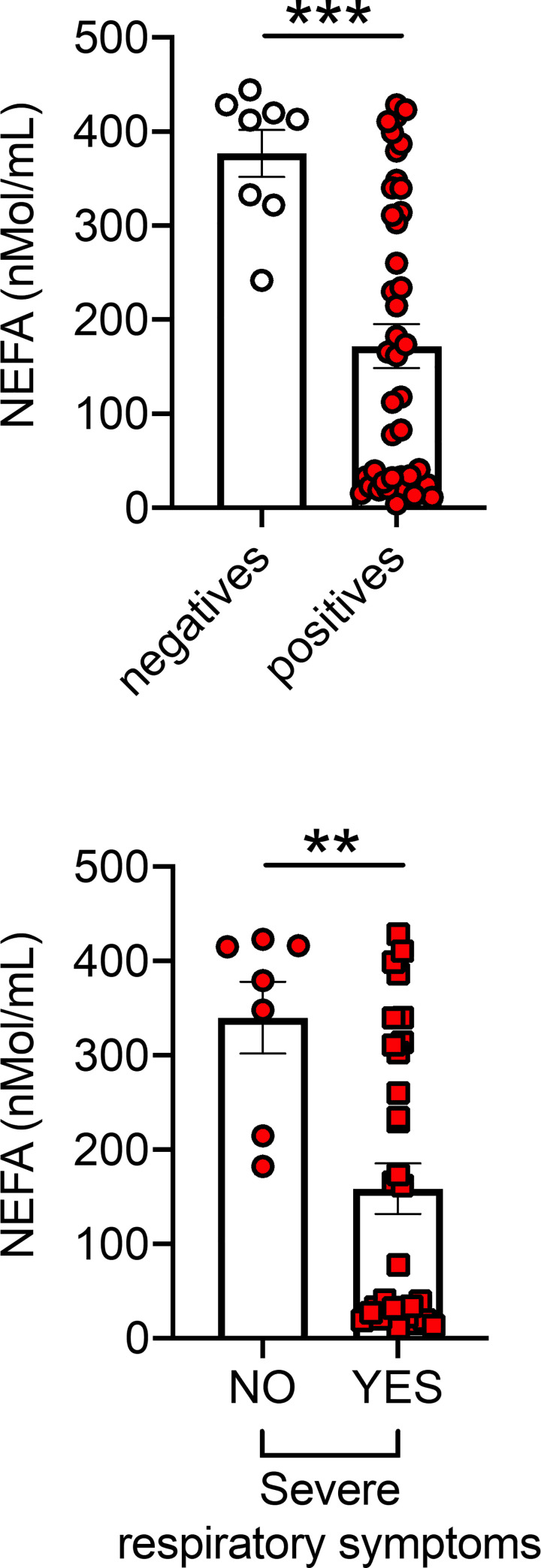
NEFA are lower in positive *versus* negative individuals. **Top.** NEFA in serum samples of participants tested negative and positive. **Bottom.** NEFA in serum samples of COVID-19 positive patients with (n = 31) or without (n = 7) severe respiratory symptoms (as defined in Fig 1). **p<0.01, ***p<0.001.

Conversely, and very interestingly, serum levels of NEFA (nonesterified fatty acids) were found lower in positive *versus* negative individuals and lower in patients with severe respiratory symptoms as compared to those with no symptoms (Fig 3). No significant differences were observed in NEFA levels between <65-year old and >65-year old positive individuals with severe respiratory symptoms (128±27 and 262±48, p = 0.19). These results could be explained by the recently published findings that the receptor binding domain of the Spike protein is physically and tightly bound to an essential fatty acid, the linoleic acid, leading to a locked Spike conformation and reduced ACE2 interaction, at least *in vitro* [48].

### SAA, CRP, and ferritin are positively associated with BMI and negatively associated with Spike-specific IgG

**Fig 4:**
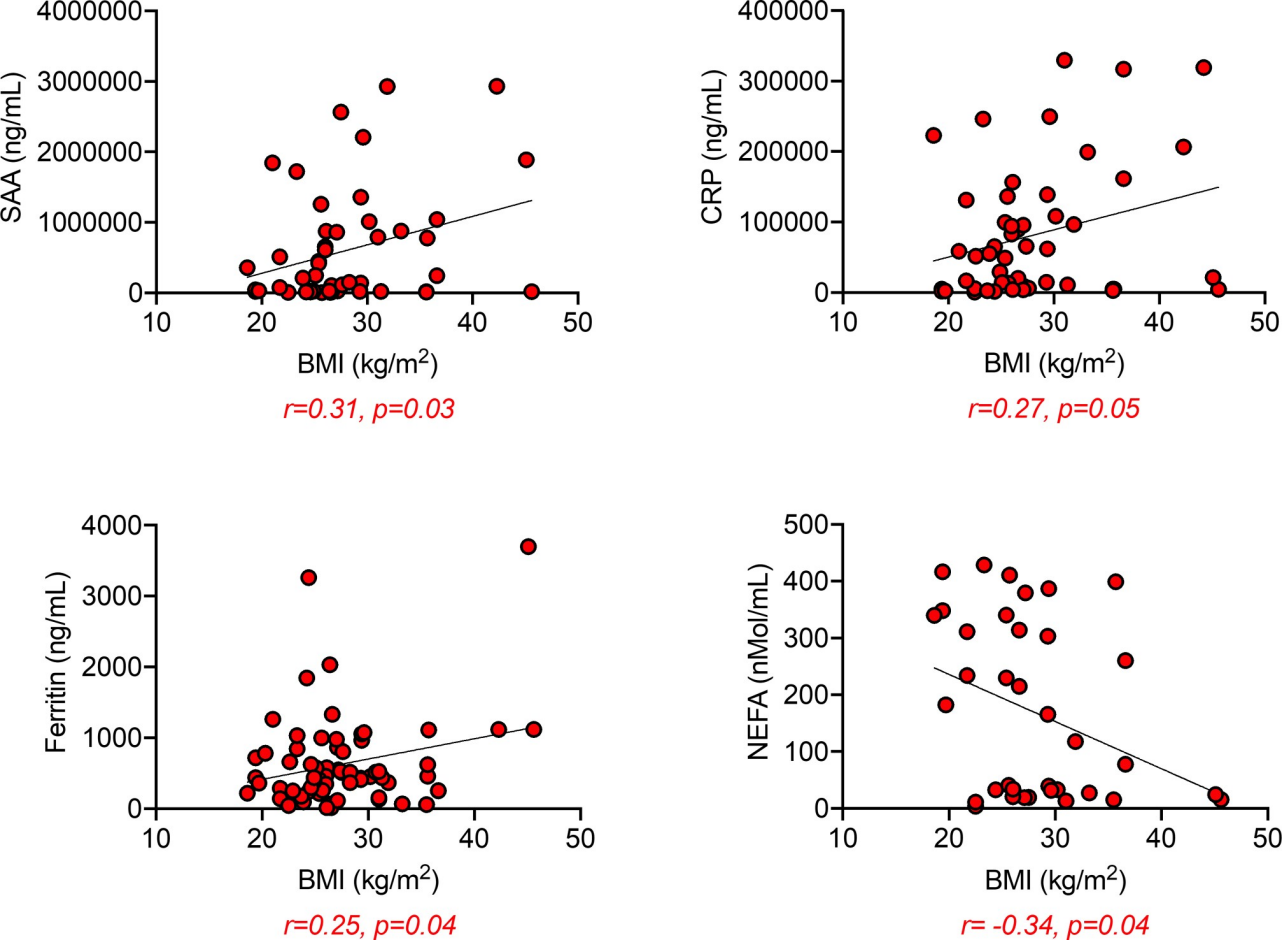
SAA, CRP, and ferritin are positively associated, whereas NEFA are negatively associated with BMI. Correlations of SAA, CRP and ferritin with BMI. Pearson’s regression coefficients and p values are indicated at the bottom of each figure.

When we performed correlation analyses between SAA, CRP, ferritin, and NEFA with BMI in positive individuals, we found, as expected, positive associations with SAA, CRP, and ferritin, and negative associations with NEFA (Fig 4).

Correlations between serum pro-inflammatory and metabolic markers in the positive individuals are shown in Table 2.

**Table 2:** Correlations between serum pro-inflammatory and metabolic markers in the positive participants.

Correlation	Person’s r	P-value
SAA & ferritin	0.43	**0.0029**
SAA & CRP	0.52	**0.0005**
SAA & NEFA	-0.41	**0.02**
Ferritin & CRP	0.05	0.73
Ferritin & NEFA	-0.11	0.05
CRP & NEFA	-0.30	0.06

In bold are indicated the correlations that are significant

**Fig 5:**
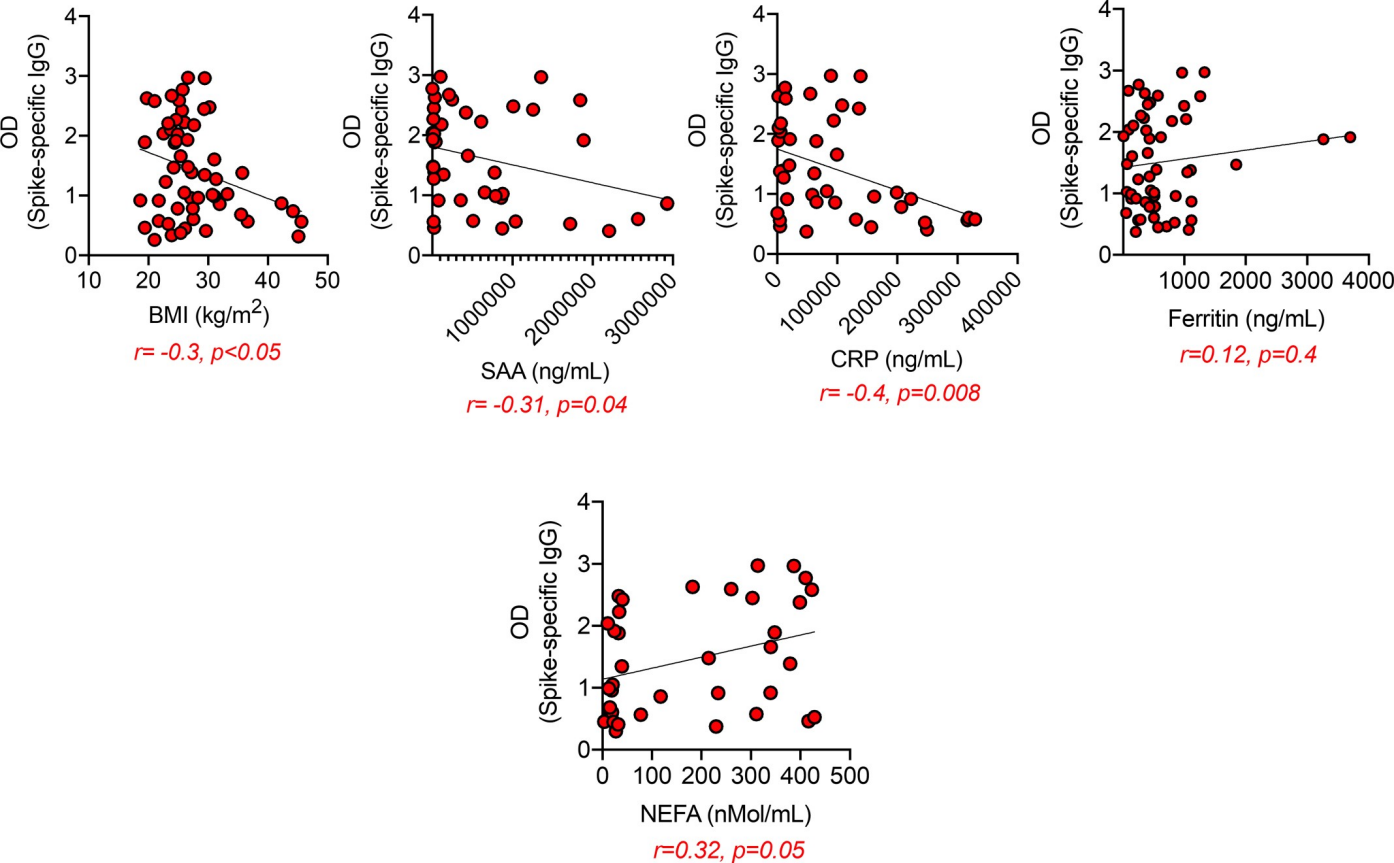
Spike-specific IgG are negatively associated with SAA and CRP and positively associated with NEFA. Correlations of Spike-specific IgG antibodies with BMI, SAA, CRP, ferritin and NEFA. Pearson’s regression coefficients and p values are indicated at the bottom of each figure.

Moreover, Spike-specific IgG levels were negatively associated with BMI, SAA, and CRP (Fig 5, top), and positively associated with NEFA (Fig 5, bottom).

## Discussion

To our knowledge, this is the first study of immunological and inflammatory profiles of COVID-19 obese patients. Although this is a rapidly evolving research field, and it has already been shown that individuals with a confirmed PCR diagnosis of infection develop antibodies against the Spike protein [49], how obesity may affect the secretion of SARS-CoV-2-specific IgG is poorly elucidated. Results herein show that serum levels of SARS-CoV-2 IgG antibodies are negatively associated with BMI in COVID-19 patients. This result is consistent with the knowledge that obesity is an inflammatory condition associated with inflammaging [1] and metaflammation [50] both of which are negatively associated with a functional immune system [51]. Another result from the present study is the negative association of SARS-CoV-2 IgG antibodies with pulmonary inflammatory markers (SAA, CRP, ferritin) in our cohort of COVID-19 patients. These are major inflammatory mediators and markers of inflammatory lung injury in patients with catastrophic acute respiratory distress syndrome, which is a primary consequence of COVID-19. SAA, CRP and ferritin are known to induce a cascade of pro-inflammatory events leading to the secretion of additional inflammation markers that contribute to the exacerbation of local and systemic inflammation resulting in dysfunctional B cells. In particular, SAA has been shown to induce secretion of several pro-inflammatory mediators by macrophages, such as IL-1β, TNF-α, IL-1RA, IL-8 [52, 53], and IL-33 [54], through activation of NF-kB and IRF7. Similar to SAA, CRP also induces secretion of pro-inflammatory cytokines (IL-6, IL-1β, TNF-α) [55] and chemokines (CCL2, CCL3, CCL4) [56] by monocytes and macrophages, whereas ferritin induces IL-1β and IL-12p70 in macrophages [57]. No direct effects of SAA, CRP and ferritin on B cells have been reported so far.

Our previously published work has shown that obesity decreases the serum antibody response to the influenza vaccine in young and elderly individuals [25] and increases autoimmune antibodies’ secretion [29, 58, 59]. We have shown that the serum concentration of leptin, the hormone secreted by the AT [39], correlates with the amount of body fat and BMI [60]. This may be at least one molecular mechanism involved in the reduced B cell function in individuals with obesity, as it induces the secretion of pro-inflammatory cytokines (IL-6/TNF-α) in human peripheral blood B cells through activation of JAK2/STAT3 and p38MAPK/ERK1/2 signaling pathways [61, 62]. Leptin also induces intrinsic B cell inflammation as measured by mRNA expression of several markers associated with immunosenescence [63]. Importantly, the expression of these markers in B cells before in vivo/in vitro stimulation negatively correlates with the same B cells’ response after stimulation [64].

We have previously shown that obesity also increases blood frequencies of the subset of double negative (DN) B cells (CD19+CD27-IgD-) [25, 59] which represents the most inflammatory B cell subset. Frequencies of DN B cells also increase in the blood of individuals with inflammatory conditions and diseases. These include aging [65–67], autoimmune diseases such as Systemic Lupus Erythematosus [68–70], Rheumatoid Arthritis [71], Sjogren’s disease [72], Multiple Sclerosis [73], Alzheimer’s disease [74], and pemphigus [75]. An increase of DN B cells has also been reported in the blood of COVID-19 patients and is associated with anti-viral antibody responses and poor clinical outcomes, as recently shown [76]. DN B cells secrete pro-inflammatory mediators that contribute to dysfunctional humoral responses and pathogenic autoantibodies that, instead of targeting disease-causing viruses, target infected individuals’ tissues. Anti-phospholipids, anti-type-I interferons, anti-nuclear antibodies, and Rheumatoid Factor have been found in a large percentage of COVID-19 patients and linked to severe disease as they may inactivate critical components of the anti-viral response [77]. It is also likely, and important to demonstrate that the neutralizing antibodies in COVID-19 patients may carry autoimmune specificity, as recently shown for broadly neutralizing antibodies against conserved domains in the influenza hemagglutinin with autoreactivity against tissue antigens previously not identified as autoantigens [78]. Overall, these findings support that the SARS-CoV-2 infection, similar to influenza, may induce self-tolerance breakdown to a variety of autoantigens, with self-tolerance breakdown already occurring in obese individuals. It is also likely that tissue failure following dissemination of the virus through the blood can induce cell death and release of intracellular antigens not known as autoantigens, in addition to those already released in the AT for mechanisms like hypoxia and consequent cell death, as we have previously demonstrated [29]. It has also recently been postulated that antibody-dependent enhancement (ADE) may exacerbate the COVID-19 disease, as previously demonstrated with dengue [79] and MERS infections [80]. However, clear evidence of ADE as a cause of severe SARS-CoV-2 infection has not yet been demonstrated. ADE occurs when antibodies are secreted in low amounts or when antibodies target but not neutralize a virus. For example, they do not block viral entry, thus facilitating Fc-receptor-mediated endocytosis of the virus and enhanced viral replication, leading to massive inflammatory responses. The quality of the antibody response is extremely relevant not only in COVID-19 patients with obesity but in every individual for future vaccination campaigns to prevent SARS-CoV-2 infection and COVID-19-associated complications in this population that is likely to be among the first to benefit from vaccination.

## Limitations of this study

There are at least two limitations in our study. First, the number of individuals recruited is limited. We are planning to expand this cohort in the future and with additional markers. Second, this study, although straightforward, only shows associations and not mechanisms for the associations. Our future studies will address possibilities after in-depth approaches.

## References

[1] Franceschi C, Bonafe M, Valensin S, Olivieri F, De Luca M, Ottaviani E, et al. Inflamm-aging. An evolutionary perspective on immunosenescence. Ann N Y Acad Sci. 2000;908:244–54. 10.1111/j.1749-6632.2000.tb06651.x . 10911963

[2] Mueller AL, McNamara MS, Sinclair DA. Why does COVID-19 disproportionately affect older people? Aging (Albany NY). 2020;12(10):9959–81. 10.18632/aging.103344 32470948 PMC7288963

[3] Frasca D, Diaz A, Romero M, Garcia D, Blomberg BB. B Cell Immunosenescence. Annu Rev Cell Dev Biol. 2020;36:551–74. 10.1146/annurev-cellbio-011620-034148 . 33021823 PMC8060858

[4] Newton AH, Cardani A, Braciale TJ. The host immune response in respiratory virus infection: balancing virus clearance and immunopathology. Semin Immunopathol. 2016;38(4):471–82. 10.1007/s00281-016-0558-0 26965109 PMC4896975

[5] Casadevall A, Pirofski LA. The convalescent sera option for containing COVID-19. J Clin Invest. 2020;130(4):1545–8. 10.1172/JCI138003 32167489 PMC7108922

[6] Duan K, Liu B, Li C, Zhang H, Yu T, Qu J, et al. Effectiveness of convalescent plasma therapy in severe COVID-19 patients. Proc Natl Acad Sci U S A. 2020;117(17):9490–6. 10.1073/pnas.2004168117 32253318 PMC7196837

[7] Langhi DM, Santis GC, Bordin JO. COVID-19 convalescent plasma transfusion. Hematol Transfus Cell Ther. 2020;42(2):113–5. 10.1016/j.htct.2020.04.003 32313872 PMC7164882

[8] Apovian CM, Gokce N. Obesity and cardiovascular disease. Circulation. 2012;125(9):1178–82. 10.1161/CIRCULATIONAHA.111.022541 22392865 PMC3693443

[9] Casas R, Sacanella E, Estruch R. The immune protective effect of the Mediterranean diet against chronic low-grade inflammatory diseases. Endocr Metab Immune Disord Drug Targets. 2014;14(4):245–54. 10.2174/1871530314666140922153350 25244229 PMC4443792

[10] Hass DJ, Brensinger CM, Lewis JD, Lichtenstein GR. The impact of increased body mass index on the clinical course of Crohn’s disease. Clin Gastroenterol Hepatol. 2006;4(4):482–8. 10.1016/j.cgh.2005.12.015 . 16616354

[11] Hotamisligil GS. Inflammation and metabolic disorders. Nature. 2006;444(7121):860–7. 10.1038/nature05485 . 17167474

[12] Johnson AM, Olefsky JM. The origins and drivers of insulin resistance. Cell. 2013;152(4):673–84. 10.1016/j.cell.2013.01.041 . 23415219

[13] Renehan AG, Tyson M, Egger M, Heller RF, Zwahlen M. Body-mass index and incidence of cancer: a systematic review and meta-analysis of prospective observational studies. Lancet. 2008;371(9612):569–78. 10.1016/S0140-6736(08)60269-X . 18280327

[14] Setty AR, Curhan G, Choi HK. Obesity, waist circumference, weight change, and the risk of psoriasis in women: Nurses’ Health Study II. Arch Intern Med. 2007;167(15):1670–5. 10.1001/archinte.167.15.1670 . 17698691

[15] Shoelson SE, Lee J, Goldfine AB. Inflammation and insulin resistance. J Clin Invest. 2006;116(7):1793–801. 10.1172/JCI29069 16823477 PMC1483173

[16] Chiappetta S, Sharma AM, Bottino V, Stier C. COVID-19 and the role of chronic inflammation in patients with obesity. Int J Obes (Lond). 2020;44(8):1790–2. 10.1038/s41366-020-0597-4 32409680 PMC7224343

[17] Rottoli M, Bernante P, Belvedere A, Balsamo F, Garelli S, Giannella M, et al. How important is obesity as a risk factor for respiratory failure, intensive care admission and death in hospitalised COVID-19 patients? Results from a single Italian centre. Eur J Endocrinol. 2020;183(4):389–97. 10.1530/EJE-20-0541 . 32674071 PMC9494325

[18] Simonnet A, Chetboun M, Poissy J, Raverdy V, Noulette J, Duhamel A, et al. High Prevalence of Obesity in Severe Acute Respiratory Syndrome Coronavirus-2 (SARS-CoV-2) Requiring Invasive Mechanical Ventilation. Obesity (Silver Spring). 2020;28(7):1195–9. 10.1002/oby.22831 32271993 PMC7262326

[19] van der Voort PHJ, Moser J, Zandstra DF, Muller Kobold AC, Knoester M, Calkhoven CF, et al. Leptin levels in SARS-CoV-2 infection related respiratory failure: A cross-sectional study and a pathophysiological framework on the role of fat tissue. Heliyon. 2020;6(8):e04696. 10.1016/j.heliyon.2020.e04696 32844126 PMC7439829

[20] Ritter A, Kreis NN, Louwen F, Yuan J. Obesity and COVID-19: Molecular Mechanisms Linking Both Pandemics. Int J Mol Sci. 2020;21(16). 10.3390/ijms21165793 32806722 PMC7460849

[21] Lighter J, Phillips M, Hochman S, Sterling S, Johnson D, Francois F, et al. Obesity in Patients Younger Than 60 Years Is a Risk Factor for COVID-19 Hospital Admission. Clin Infect Dis. 2020;71(15):896–7. 10.1093/cid/ciaa415 32271368 PMC7184372

[22] Falagas ME, Kompoti M. Obesity and infection. Lancet Infect Dis. 2006;6(7):438–46. 10.1016/S1473-3099(06)70523-0 . 16790384

[23] Karlsson EA, Beck MA. The burden of obesity on infectious disease. Exp Biol Med (Maywood). 2010;235(12):1412–24. 10.1258/ebm.2010.010227 . 21127339

[24] O’Shea D, Corrigan M, Dunne MR, Jackson R, Woods C, Gaoatswe G, et al. Changes in human dendritic cell number and function in severe obesity may contribute to increased susceptibility to viral infection. Int J Obes (Lond). 2013;37(11):1510–3. 10.1038/ijo.2013.16 . 23439322

[25] Frasca D, Ferracci F, Diaz A, Romero M, Lechner S, Blomberg BB. Obesity decreases B cell responses in young and elderly individuals. Obesity (Silver Spring). 2016;24(3):615–25. 10.1002/oby.21383 26857091 PMC4769695

[26] Ovsyannikova IG, White SJ, Larrabee BR, Grill DE, Jacobson RM, Poland GA. Leptin and leptin-related gene polymorphisms, obesity, and influenza A/H1N1 vaccine-induced immune responses in older individuals. Vaccine. 2014;32(7):881–7. 10.1016/j.vaccine.2013.12.009 24360890 PMC3922536

[27] Sheridan PA, Paich HA, Handy J, Karlsson EA, Hudgens MG, Sammon AB, et al. Obesity is associated with impaired immune response to influenza vaccination in humans. Int J Obes (Lond). 2012;36(8):1072–7. 10.1038/ijo.2011.208 22024641 PMC3270113

[28] George MD, Baker JF. The Obesity Epidemic and Consequences for Rheumatoid Arthritis Care. Curr Rheumatol Rep. 2016;18(1):6. 10.1007/s11926-015-0550-z 26739963 PMC4809046

[29] Frasca D, Diaz A, Romero M, Thaller S, Blomberg BB. Secretion of autoimmune antibodies in the human subcutaneous adipose tissue. PLoS One. 2018;13(5):e0197472. 10.1371/journal.pone.0197472 29768501 PMC5955545

[30] Grant RW, Dixit VD. Adipose tissue as an immunological organ. Obesity (Silver Spring). 2015;23(3):512–8. 10.1002/oby.21003 25612251 PMC4340740

[31] Franssen FM, O’Donnell DE, Goossens GH, Blaak EE, Schols AM. Obesity and the lung: 5. Obesity and COPD. Thorax. 2008;63(12):1110–7. 10.1136/thx.2007.086827 . 19020276

[32] Murugan AT, Sharma G. Obesity and respiratory diseases. Chron Respir Dis. 2008;5(4):233–42. 10.1177/1479972308096978 . 19029235

[33] Kruglikov IL, Scherer PE. The role of adipocytes and adipocyte-like cells in the severity of COVID-19 infections. Obesity (Silver Spring). 2020; 10.1002/oby.22856 . 32339391 PMC7267593

[34] Tan L, Wang Q, Zhang D, Ding J, Huang Q, Tang YQ, et al. Lymphopenia predicts disease severity of COVID-19: a descriptive and predictive study. Signal Transduct Target Ther. 2020;5(1):33. 10.1038/s41392-020-0148-4 32296069 PMC7100419

[35] Arunachalam PS, Wimmers F, Mok CKP, Perera R, Scott M, Hagan T, et al. Systems biological assessment of immunity to mild versus severe COVID-19 infection in humans. Science. 2020;369(6508):1210–20. 10.1126/science.abc6261 . 32788292 PMC7665312

[36] Mathew D, Giles JR, Baxter AE, Oldridge DA, Greenplate AR, Wu JE, et al. Deep immune profiling of COVID-19 patients reveals distinct immunotypes with therapeutic implications. Science. 2020;369(6508). 10.1126/science.abc8511 32669297 PMC7402624

[37] Woodruff MC, Ramonell RP, Nguyen DC, Cashman KS, Saini AS, Haddad NS, et al. Extrafollicular B cell responses correlate with neutralizing antibodies and morbidity in COVID-19. Nat Immunol. 2020. 10.1038/s41590-020-00814-z . 33028979 PMC7739702

[38] Iikuni N, Lam QL, Lu L, Matarese G, La Cava A. Leptin and Inflammation. Curr Immunol Rev. 2008;4(2):70–9. 10.2174/157339508784325046 20198122 PMC2829991

[39] Zhang Y, Proenca R, Maffei M, Barone M, Leopold L, Friedman JM. Positional cloning of the mouse obese gene and its human homologue. Nature. 1994;372(6505):425–32. 10.1038/372425a0 . 7984236

[40] Cheng L, Yang JZ, Bai WH, Li ZY, Sun LF, Yan JJ, et al. Prognostic value of serum amyloid A in patients with COVID-19. Infection. 2020;48(5):715–22. 10.1007/s15010-020-01468-7 32734556 PMC7391472

[41] Li H, Xiang X, Ren H, Xu L, Zhao L, Chen X, et al. Serum Amyloid A is a biomarker of severe Coronavirus Disease and poor prognosis. J Infect. 2020;80(6):646–55. 10.1016/j.jinf.2020.03.035 32277967 PMC7141628

[42] Luo X, Zhou W, Yan X, Guo T, Wang B, Xia H, et al. Prognostic value of C-reactive protein in patients with COVID-19. Clin Infect Dis. 2020. 10.1016/j.jcv.2020.104370 32445579 PMC7314209

[43] Manson JJ, Crooks C, Naja M, Ledlie A, Goulden B, Liddle T, et al. COVID-19-associated hyperinflammation and escalation of patient care: a retrospective longitudinal cohort study. Lancet Rheumatol. 2020;2(10):e594–e602. 10.1016/S2665-9913(20)30275-7 32864628 PMC7442426

[44] Velavan TP, Meyer CG. Mild versus severe COVID-19: Laboratory markers. Int J Infect Dis. 2020;95:304–7. 10.1016/j.ijid.2020.04.061 32344011 PMC7194601

[45] Wang L. C-reactive protein levels in the early stage of COVID-19. Med Mal Infect. 2020;50(4):332–4. 10.1016/j.medmal.2020.03.007 32243911 PMC7146693

[46] Gomez-Pastora J, Weigand M, Kim J, Wu X, Strayer J, Palmer AF, et al. Hyperferritinemia in critically ill COVID-19 patients—Is ferritin the product of inflammation or a pathogenic mediator? Clin Chim Acta. 2020;509:249–51. 10.1016/j.cca.2020.06.033 32579952 PMC7306200

[47] Zhou F, Yu T, Du R, Fan G, Liu Y, Liu Z, et al. Clinical course and risk factors for mortality of adult inpatients with COVID-19 in Wuhan, China: a retrospective cohort study. Lancet. 2020;395(10229):1054–62. 10.1016/S0140-6736(20)30566-3 32171076 PMC7270627

[48] Toelzer C, Gupta K, Yadav SKN, Borucu U, Davidson AD, Kavanagh Williamson M, et al. Free fatty acid binding pocket in the locked structure of SARS-CoV-2 spike protein. Science. 2020;370(6517):725–30. 10.1126/science.abd3255 . 32958580 PMC8050947

[49] Seow J, Graham C, Merrick B, Acors S, Pickering S, Steel KJA, et al. Longitudinal observation and decline of neutralizing antibody responses in the three months following SARS-CoV-2 infection in humans. Nat Microbiol. 2020;5(12):1598–607. 10.1038/s41564-020-00813-8 . 33106674 PMC7610833

[50] Hotamisligil GS. Inflammation, metaflammation and immunometabolic disorders. Nature. 2017;542(7640):177–85. 10.1038/nature21363 . 28179656

[51] Ahima RS. Connecting obesity, aging and diabetes. Nat Med. 2009;15(9):996–7. 10.1038/nm0909-996 . 19734871

[52] Furlaneto CJ, Campa A. A novel function of serum amyloid A: a potent stimulus for the release of tumor necrosis factor-alpha, interleukin-1beta, and interleukin-8 by human blood neutrophil. Biochem Biophys Res Commun. 2000;268(2):405–8. 10.1006/bbrc.2000.2143 . 10679217

[53] Patel H, Fellowes R, Coade S, Woo P. Human serum amyloid A has cytokine-like properties. Scand J Immunol. 1998;48(4):410–8. 10.1046/j.1365-3083.1998.00394.x . 9790312

[54] Sun L, Zhu Z, Cheng N, Yan Q, Ye RD. Serum amyloid A induces interleukin-33 expression through an IRF7-dependent pathway. Eur J Immunol. 2014;44(7):2153–64. 10.1002/eji.201344310 24777946 PMC4118754

[55] Ballou SP, Lozanski G. Induction of inflammatory cytokine release from cultured human monocytes by C-reactive protein. Cytokine. 1992;4(5):361–8. 10.1016/1043-4666(92)90079-7 . 1420997

[56] Montecucco F, Steffens S, Burger F, Pelli G, Monaco C, Mach F. C-reactive protein (CRP) induces chemokine secretion via CD11b/ICAM-1 interaction in human adherent monocytes. J Leukoc Biol. 2008;84(4):1109–19. 10.1189/jlb.0208123 . 18591415

[57] Ruscitti P, Di Benedetto P, Berardicurti O, Panzera N, Grazia N, Lizzi AR, et al. Pro-inflammatory properties of H-ferritin on human macrophages, ex vivo and in vitro observations. Sci Rep. 2020;10(1):12232. 10.1038/s41598-020-69031-w 32699419 PMC7376151

[58] Frasca D, Diaz A, Romero M, Garcia D, Jayram D, Thaller S, et al. Identification and Characterization of Adipose Tissue-Derived Human Antibodies With "Anti-self" Specificity. Front Immunol. 2020;11:392. 10.3389/fimmu.2020.00392 32184790 PMC7058997

[59] Frasca D, Diaz A, Romero M, Thaller S, Blomberg BB. Metabolic requirements of human pro-inflammatory B cells in aging and obesity. PLoS One. 2019;14(7):e0219545. 10.1371/journal.pone.0219545 31287846 PMC6615614

[60] La Cava A, Matarese G. The weight of leptin in immunity. Nat Rev Immunol. 2004;4(5):371–9. 10.1038/nri1350 . 15122202

[61] Agrawal S, Gollapudi S, Su H, Gupta S. Leptin activates human B cells to secrete TNF-alpha, IL-6, and IL-10 via JAK2/STAT3 and p38MAPK/ERK1/2 signaling pathway. J Clin Immunol. 2011;31(3):472–8. 10.1007/s10875-010-9507-1 21243519 PMC3132280

[62] Gupta S, Agrawal S, Gollapudi S. Increased activation and cytokine secretion in B cells stimulated with leptin in aged humans. Immun Ageing. 2013;10(1):3. 10.1186/1742-4933-10-3 23343052 PMC3557206

[63] Frasca D, Diaz A, Romero M, Blomberg BB. Leptin induces immunosenescence in human B cells. Cell Immunol. 2020;348:103994. 10.1016/j.cellimm.2019.103994 31831137 PMC7002206

[64] Frasca D, Diaz A, Romero M, Landin AM, Blomberg BB. High TNF-alpha levels in resting B cells negatively correlate with their response. Exp Gerontol. 2014;54:116–22. 10.1016/j.exger.2014.01.004 24440385 PMC3989457

[65] Colonna-Romano G, Bulati M, Aquino A, Pellicano M, Vitello S, Lio D, et al. A double-negative (IgD-CD27-) B cell population is increased in the peripheral blood of elderly people. Mech Ageing Dev. 2009;130(10):681–90. 10.1016/j.mad.2009.08.003 . 19698733

[66] Frasca D, Diaz A, Romero M, Blomberg BB. Human peripheral late/exhausted memory B cells express a senescent-associated secretory phenotype and preferentially utilize metabolic signaling pathways. Exp Gerontol. 2017;87(Pt A):113–20. 10.1016/j.exger.2016.12.001 . 27931848

[67] Nevalainen T, Autio A, Kummola L, Salomaa T, Junttila I, Jylha M, et al. CD27- IgD- B cell memory subset associates with inflammation and frailty in elderly individuals but only in males. Immun Ageing. 2019;16:19. 10.1186/s12979-019-0159-6 31423147 PMC6693136

[68] Jenks SA, Cashman KS, Zumaquero E, Marigorta UM, Patel AV, Wang X, et al. Distinct Effector B Cells Induced by Unregulated Toll-like Receptor 7 Contribute to Pathogenic Responses in Systemic Lupus Erythematosus. Immunity. 2018;49(4):725–39 e6. 10.1016/j.immuni.2018.08.015 30314758 PMC6217820

[69] Wang S, Wang J, Kumar V, Karnell JL, Naiman B, Gross PS, et al. IL-21 drives expansion and plasma cell differentiation of autoreactive CD11c(hi)T-bet(+) B cells in SLE. Nat Commun. 2018;9(1):1758. 10.1038/s41467-018-03750-7 29717110 PMC5931508

[70] Wehr C, Eibel H, Masilamani M, Illges H, Schlesier M, Peter HH, et al. A new CD21low B cell population in the peripheral blood of patients with SLE. Clin Immunol. 2004;113(2):161–71. 10.1016/j.clim.2004.05.010 . 15451473

[71] Adlowitz DG, Barnard J, Biear JN, Cistrone C, Owen T, Wang W, et al. Expansion of Activated Peripheral Blood Memory B Cells in Rheumatoid Arthritis, Impact of B Cell Depletion Therapy, and Biomarkers of Response. PLoS One. 2015;10(6):e0128269. 10.1371/journal.pone.0128269 26047509 PMC4457888

[72] Saadoun D, Terrier B, Bannock J, Vazquez T, Massad C, Kang I, et al. Expansion of autoreactive unresponsive CD21-/low B cells in Sjogren’s syndrome-associated lymphoproliferation. Arthritis Rheum. 2013;65(4):1085–96. 10.1002/art.37828 23279883 PMC4479193

[73] Claes N, Fraussen J, Vanheusden M, Hellings N, Stinissen P, Van Wijmeersch B, et al. Age-Associated B Cells with Proinflammatory Characteristics Are Expanded in a Proportion of Multiple Sclerosis Patients. J Immunol. 2016;197(12):4576–83. 10.4049/jimmunol.1502448 . 27837111

[74] Martorana A, Balistreri CR, Bulati M, Buffa S, Azzarello DM, Camarda C, et al. Double negative (CD19+IgG+IgD-CD27-) B lymphocytes: a new insight from telomerase in healthy elderly, in centenarian offspring and in Alzheimer’s disease patients. Immunol Lett. 2014;162(1 Pt B):303–9. 10.1016/j.imlet.2014.06.003 . 24951896

[75] Golinski ML, Demeules M, Derambure C, Riou G, Maho-Vaillant M, Boyer O, et al. CD11c(+) B Cells Are Mainly Memory Cells, Precursors of Antibody Secreting Cells in Healthy Donors. Front Immunol. 2020;11:32. 10.3389/fimmu.2020.00032 32158442 PMC7051942

[76] Woodruff MC, Ramonell RP, Nguyen DC, Cashman KS, Saini AS, Haddad NS, et al. Extrafollicular B cell responses correlate with neutralizing antibodies and morbidity in COVID-19. Nat Immunol. 2020;21(12):1506–16. 10.1038/s41590-020-00814-z . 33028979 PMC7739702

[77] Woodruff M, Ramonell R, Lee E-H, Sanz I. Clinically identifiable autoreactivity is common in severe SARS-CoV-2 Infection. medRxiv. 2020. 10.1101/2020.10.21.2021619201/2020.04.29.20083717 32511635

[78] Khurana S, Hahn M, Klenow L, Golding H. Autoreactivity of Broadly Neutralizing Influenza Human Antibodies to Human Tissues and Human Proteins. Viruses. 2020;12(10). 10.3390/v12101140 33049994 PMC7600923

[79] Cui G, Si L, Wang Y, Zhou J, Yan H, Jiang L. Antibody-dependent enhancement (ADE) of dengue virus: Identification of the key amino acid that is vital in DENV vaccine research. J Gene Med. 2021;23(2):e3297. 10.1002/jgm.3297 . 33217097 PMC7900978

[80] Wan Y, Shang J, Sun S, Tai W, Chen J, Geng Q, et al. Molecular Mechanism for Antibody-Dependent Enhancement of Coronavirus Entry. J Virol. 2020;94(5). 10.1128/JVI.02015-19 31826992 PMC7022351

